# Plasticity of GABAA Receptors during Pregnancy and Postpartum Period: From Gene to Function

**DOI:** 10.1155/2015/170435

**Published:** 2015-08-30

**Authors:** Valentina Licheri, Giuseppe Talani, Ashish A. Gorule, Maria Cristina Mostallino, Giovanni Biggio, Enrico Sanna

**Affiliations:** ^1^Department of Life and Environmental Sciences, University of Cagliari, 09126 Cagliari, Italy; ^2^Institute of Neuroscience, National Research Council, Monserrato, 09042 Cagliari, Italy

## Abstract

Pregnancy needs complex pathways that together play a role in proper growth and protection of the fetus preventing its premature loss. Changes during pregnancy and postpartum period include the manifold machinery of neuroactive steroids that plays a crucial role in neuronal excitability by local modulation of specific inhibitory receptors: the GABAA receptors. Marked fluctuations in both blood and brain concentration of neuroactive steroids strongly contribute to GABAA receptor function and plasticity. In this review, we listed several interesting results regarding the regulation and plasticity of GABAA receptor function during pregnancy and postpartum period in rats. The increase in brain levels of neuroactive steroids during pregnancy and their sudden decrease immediately before delivery are causally related to changes in the expression/function of specific GABAA receptor subunits in the hippocampus. These data suggest that alterations in GABAA receptor expression and function may be related to neurological and psychiatric disorders associated with crucial periods in women. These findings could help to provide potential new treatments for these women's disabling syndromes.

## 1. Introduction

Pregnancy and postpartum period are crucial periods for a woman's life where many physiological changes take place, especially at the hormone levels. Several neurotransmitter systems, such as the GABAergic system, are particularly subject to such hormonal fluctuations, undergoing, in response, dramatic functional changes. It is well established that the neurotransmitter *γ*-aminobutyric acid (GABA), acting at the GABAA receptor (GABAAR), mediates its main inhibitory effect through the so-called “fast” inhibitory synaptic transmission in the mammalian central nervous system (CNS) leading to a profound influence on mood and behavior. Synaptic and nonsynaptic GABAARs are pentameric ionotropic complexes mainly formed by *α*, *β*, and another type of subunit (including *γ*, *δ*, or *ε*), with a common proportion stoichiometry of 2 : 2 : 1, respectively [1]. As widely reported, *α* and *β* subunits are currently present in all different receptor compositions; the *γ* subunit is mainly associated with GABAAR expressed in the synaptic compartment (or extrasynaptic compartment when associated with the *α*5 subunit), while *δ* is associated with receptors present at extrasynaptic level [2–4].

The synaptic receptors mediate the “phasic” component of GABAergic inhibition, while the extrasynaptic receptors mediate a sustained “tonic” form of inhibition and thereby play a key role in brain excitability in both physiological and pathological states [5, 6]. Pharmacological studies and analyses in knockout mice have shown that these receptors not only are important for mediating tonic current [7–12], but also represent physiological targets of neuroactive steroids, possess a high affinity for GABA, and are characterized by an extremely slow rate of desensitization [13, 14]. Neuroactive steroids, in a concentration range thought to be present in the extracellular space under physiological conditions, such as during pregnancy and postpartum period, selectively enhance the magnitude of tonic inhibition mediated by *δ* subunit-containing GABAARs, resulting in a decrease in network excitability [8]. 3*α*,5*α*-THP (3*α*-hydroxy-5*α*-pregnan-20-one, allopregnanolone) is one of the most studied neuroactive steroids, a pregnane neurosteroid metabolite of its precursor progesterone, generated by the sequential actions of two different enzymes, 5*α*-reductase and 3*α*-hydroxysteroid dehydrogenase [15, 16]. Due to its potent positive action exerted at GABAARs, 3*α*,5*α*-THP is among the most important endogenous positive allosteric modulators of both synaptic and extrasynaptic GABAARs [17]. Its mechanism of action consists in prolongation of the opening time of the chloride ion channel associated with GABAARs resulting in increased inhibitory neurotransmission [18, 19].

For years, several research groups have directed their studies on the effects of neuroactive steroid fluctuations during fundamental time window in female rodents such as menstrual cycle, pregnancy, and postpartum period and the consequent changes in blood and brain concentrations of neuroactive steroids that can strongly affect the expression and function of specific GABAAR subunits [20]. In this review article, we summarize a series of findings that emerged from our and other research groups related to the differential changes in the expression and function of both synaptic and extrasynaptic GABAARs in the rat hippocampus during pregnancy and after delivery.

## 2. Neuroactive Steroids and Pregnancy

In both menstrual cycle and pregnancy, the corpus luteum and the placenta, respectively, are implicated in the production of progesterone essential to maintain healthy pregnancy. Both progesterone and some of its metabolites are also increased in pregnant women [21, 22]. In addition, the activity of the enzymes responsible for the synthesis of 3*α*,5*α*-THP is increased in maternal as well as fetal tissue [23, 24], suggesting that both production and metabolism of these compounds appear to be fundamental during pregnancy.

We have shown that brain and plasma concentrations of progesterone and 3*α*,5*α*-THP are increased in pregnant rats, returning to basal values (present during the estrus phase of the menstrual cycle) immediately before delivery; values remain unchanged during the postpartum period [25]. Brain levels of 3*α*,5*α*-THP detected during pregnancy are mostly derived from circulating progesterone, although the brain has also the ability to synthesize 3*α*,5*α*-THP* de novo* from cholesterol [26] under physiologic as well as pharmacological conditions [27, 28]. However, the time course of the changes in concentrations differs substantially between progesterone and its metabolite 3*α*,5*α*-THP; the concentration of progesterone in the cerebral cortex and hippocampus, like that in plasma, peaks on day 15 of pregnancy (P15), reaching values that result 12 and 10 times higher than those measured during estrus, respectively, remaining substantially increased at P19; on the other hand, the concentrations of 3*α*,5*α*-THP in the same brain regions do not peak until P19 (208% and 194% increase in the cerebral cortex and hippocampus, resp.).

At P21, the concentrations of both progesterone and 3*α*,5*α*-THP in the brain and plasma drop down to values typical of estrus and do not change during the first 7 days after delivery [25]. Furthermore, also the activity of 5*α*-reductase or 3*α*-hydroxysteroid dehydrogenase (both enzymes required for 3*α*,5*α*-THP synthesis starting from progesterone) in the brain during pregnancy might be crucial for the regulation of steroid levels. In this regard, 17*β*-estradiol has been shown to upregulate 5*α*-reductase [29] as well as 3*α*-hydroxysteroid dehydrogenase activity [15] in the rat brain.

Thus, given that 3*α*,5*α*-THP modulates positively the function of GABAARs [30, 31], subsequent studies were undertaken to determine whether the physiological fluctuations of neuroactive steroids that occur during pregnancy and after delivery might also influence the expression of various subunits of both synaptic and extrasynaptic GABAARs in rat brain.

## 3. Expression and Function of GABAARs during Pregnancy and Postpartum Period

Our laboratory has recently studied the relationship between changes in neuroactive steroids and expression of GABAAR subunits during pregnancy and after delivery in rats, comparing the data with the basal levels measured during the estrus of estrous cycle, where the levels of pregnane steroids are low [32].

**Figure 1:**
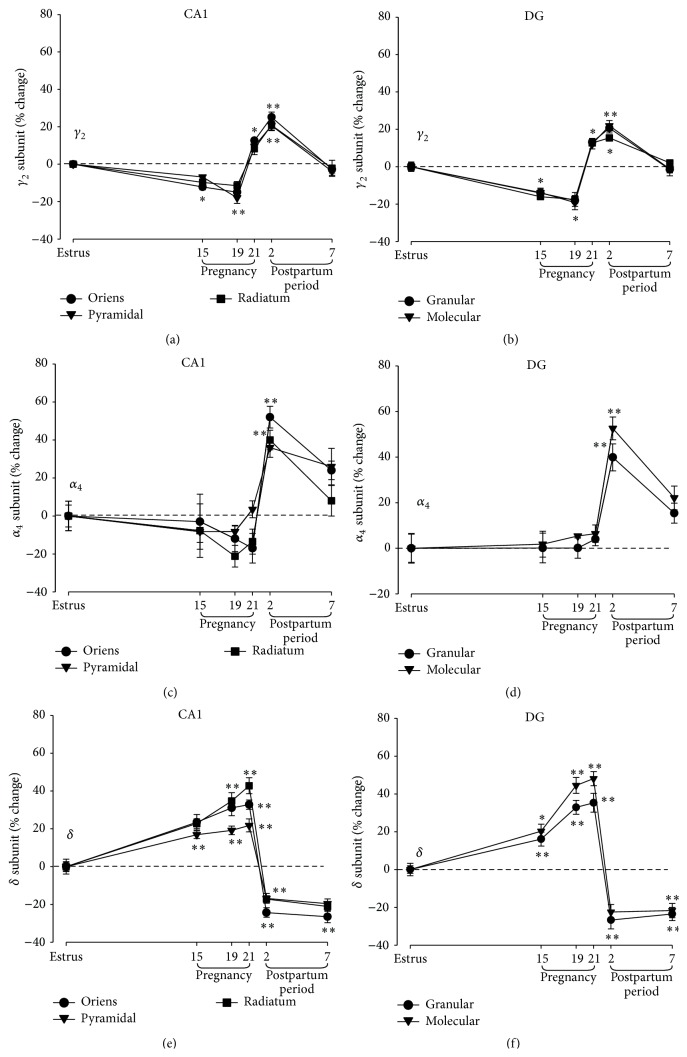
Changes in immunoreactivity for different subunits of the GABAAR in the rat hippocampus during pregnancy and after delivery. Scatter plot relative to immunoreactivity quantification of different GABAAR subunits (*γ*2, *α*4, and *δ*) evaluated in the hippocampal CA1 subregion (left panels) and dentate gyrus (right panels) during pregnancy and after delivery. Adapted from Sanna et al., 2009 [34].

*GABAAR γ2 Subunit*. In the hippocampus, the *γ*2 subunit is mainly expressed in the strata oriens and radiatum of both CA1 and CA3 subregions, in the stratum lacunosum-moleculare of CA1, and in the granule cell layer of the dentate gyrus of rats in estrus. The immunoreactivity for the *γ*2 subunit of the GABAAR in rat brain decreases progressively during pregnancy [25, 33] (Figure 1). The amount of this subunit remained unchanged until P10 was reached but was significantly reduced (by ~30%) between P15 and P19 when compared with the level apparent in control rats evaluated during estrus. In contrast, after delivery, by postnatal day 2 (PND2), the amount of the *γ*2 subunit was increased and returned to the estrus level at PND7 [20, 25, 33]. All these changes appeared similar in both the CA1 subregion and dentate gyrus (Figures 1(a) and 1(b)). Similar to the hippocampal formation, in the cerebral cortex of pregnant rats, the *γ*2 subunit goes through alike changes. Parallel to these changes, muscimol-induced Cl^−^ uptake and the potentiating effects of diazepam and 3*α*,5*α*-THP on muscimol-induced Cl^−^ uptake were also reduced during pregnancy while they were markedly increased after delivery [25, 33], returning to control values within 7 days of postnatal life.

*GABAAR α4 Subunit*. The characterization of the *α*4 subunit expression pattern revealed that this subunit appears to be distributed diffusely throughout the hippocampal formation of rats. In female rats during estrus, it is particularly abundant in the granule cell layer of the dentate gyrus and in the CA1 pyramidal cell layer as indicated by both mRNA and immunostaining evaluations [34]. In contrast to what was observed for the *γ*2 subunit, the immunoreactivity for the *α*4 subunit is unchanged in both hippocampus (Figures 1(c) and 1(d)) and cerebral cortex of pregnant rats [25, 33, 34] but it undergoes a marked increase right after delivery in the dentate gyrus granule cell layer and in the CA1 subregion of the hippocampus (Figures 1(c) and 1(d)) [34, 35].

*GABAAR δ Subunit*. As described for the *α*4 subunit, moderate levels of immunoreactivity for the *δ* subunit have been reported throughout different regions of the hippocampal formation of rats during estrus, but it appeared mainly expressed in the granule cell layer as well as in the molecular layer of the dentate gyrus [34, 36]. During pregnancy and, particularly, between P15 and P21, the *δ* subunit immunoreactivity increased progressively in the dentate gyrus but also in CA1 subregion when compared with rats in estrus [34]. After delivery, there is reduction of the expression of the *δ* subunit that is still apparent 7 days after delivery in the granular and molecular layers of the dentate gyrus as well as in the CA1 subregion [34].

## 4. GABAAR-Mediated Phasic Inhibition during Pregnancy and Postpartum Period

All the changes in GABAAR expression observed during pregnancy and postpartum period in rats are expected to reflect parallel modifications in the function of both synaptic and extrasynaptic GABAARs. To further assess such possibility, we studied phasic GABAergic inhibition by electrophysiological voltage-clamp recordings in granule cells of the dentate gyrus, evaluating the spontaneous inhibitory postsynaptic currents (sIPSCs).

During pregnancy (P15, P19) and 2 days after delivery, the basal kinetic properties, such as amplitude, decay time, area, and frequency, of IPSCs recorded from dentate gyrus granule cells were not significantly different from those detected in rats in estrus [34]. In a separate set of experiments, we evaluated whether pregnancy or delivery might affect the sensitivity of synaptic GABAARs to the action of various allosteric modulators such as neuroactive steroids and benzodiazepines. Perfusion of 3*α*,5*α*-THP (1 *μ*M) caused a marked increase in the decay time constant, amplitude, and area of GABAAR-mediated sIPSCs but this effect did not differ between rats tested at P19 and those recorded during estrus [34]. Also the modulatory effect of the benzodiazepine lorazepam (3 *μ*M) was slightly reduced, without reaching statistical significance, in rats at P19 compared with those in estrus. The increased expression of the *α*4 and *γ*2 subunits of the GABAAR in the hippocampus 2 days after delivery could promote a parallel increase in function of GABAAR containing those subunits. We thus tested the action of Ro15-4513 on sIPSCs in granule cells of the dentate gyrus. The pharmacological profile of Ro15-4513 is very much influenced by the subunit composition; this drug is an inverse agonist on GABAAR formed by *α*1, *α*2, *α*3, or *α*5 subunit together with the *β* and *γ*2 subunit [1], but it behaves as a positive modulator in receptors formed by *α*4, *β*, and *γ*2 subunits [37, 38]. In our experiments, Ro15-4513 (3 *μ*M) reduced the decay time constant of sIPSCs in granule cells of rats in estrus or at P19, but it increased this parameter in granule cells of rats when tested 2 days after delivery, with the increased expression of the *α*4 subunit at this time [34].

## 5. GABAAR-Mediated Tonic Inhibition during Pregnancy and Postpartum Period

As mentioned in the previous section, in addition to synaptic GABAARs, which are responsible for phasic inhibition, granule cells of the dentate gyrus in adult rats express a high concentration of extrasynaptic receptors that are formed mainly by the combination of *α*4, *β*_*n*_, and *δ* subunits [39], which are the first candidate for the tonic conductance of GABAergic inhibition in this cell population. Other reports highlighted the presence of a small population of extrasynaptic receptors formed by *α*1, *β*_*n*_, and *δ* that are selectively localized onto GABAergic interneurons in the molecular layer of the dentate gyrus [3] or formed by *α*5 subunit, which are present in the CA1/CA3 region and dentate gyrus [4, 36, 40].

**Figure 2:**
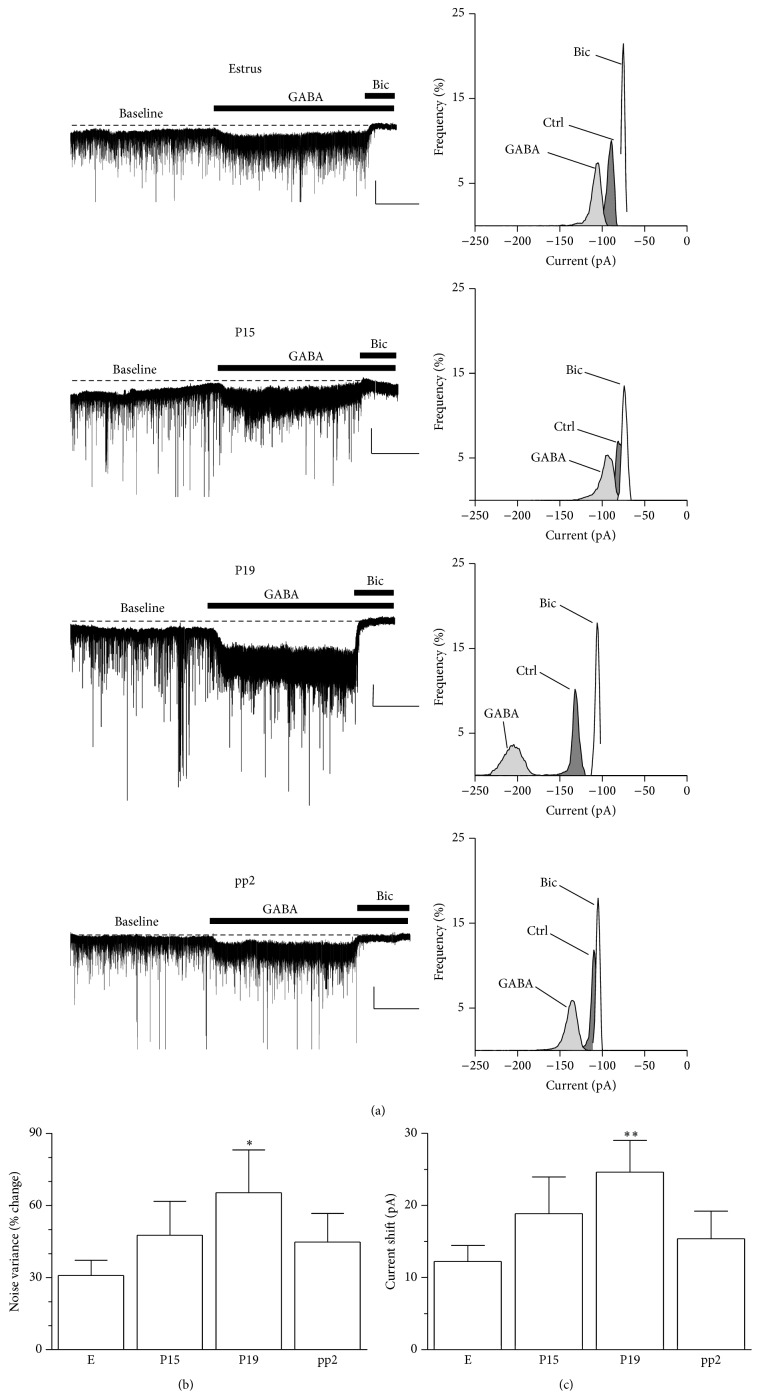
Changes in GABAergic tonic current in granule cells of the dentate gyrus during pregnancy and after delivery. (a) Representative traces (left) and all-point histogram (right) relative to GABAergic currents recorded in the whole-cell mode from granule cells of the dentate gyrus of hippocampal slices isolated from rats in estrus, at day P15 or P19, or at pp2. The effect of GABA (5 *μ*M) and bicuculline (30 *μ*M) is reported. Calibration: 50 pA, 2 min. (b, c) Bar graphs summarizing the changes in noise variance (b) and holding current (c) induced by the application of exogenous GABA in experiments similar to those shown in (a). Adapted from Sanna et al., 2009 [34].

**Figure 3:**
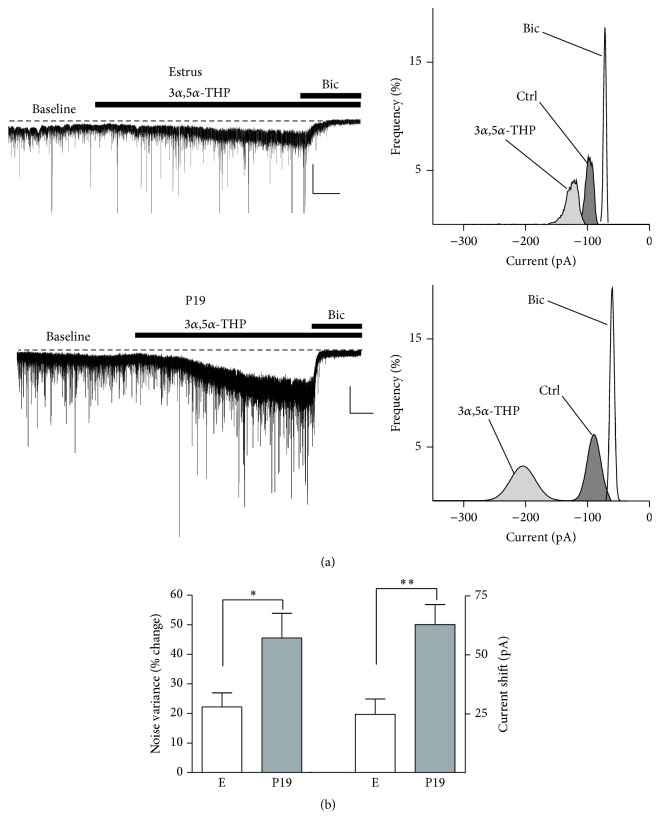
Modulatory action of 3*α*,5*α*-THP on tonic GABAergic current in granule cells of the dentate gyrus during pregnancy. (a) Representative traces (left) and all-points histogram (right) of GABAergic currents recorded from granule cells in hippocampal slices from rats in estrus or at P19. The effect of 3*α*,5*α*-THP (1 *μ*M) and bicuculline (30 *μ*M) is reported. (b) Bar graph summarizing the percentage changes in noise variance (left* y*-axes) and holding current (right* y-*axes) induced by 3*α*,5*α*-THP in experiments similar to those shown in (a). Adapted from Sanna et al., 2009 [34].

This peculiar mechanism of the GABAergic inhibition plays a key role in those physiological conditions, such as pregnancy and postpartum period, where GABAAR subunits, implicated in the mediation of tonic current, undergo marked change in expression. We recorded GABAergic tonic currents in granule cells of the dentate gyrus in hippocampal acute slices [34]. Bath application of GABA (5 *μ*M) for 5 min stimulates high-affinity extrasynaptic GABAARs increasing the current noise variance and the negative shift in the holding current with respect to baseline. This effect is evident in slices from rats in estrus although, consistent with the increase in the expression of the *δ* subunit during pregnancy, we found that the effect of GABA on tonic current parameters is greater when this agonist is perfused in slices from pregnant rats at P15, and it reaches a maximal effect at P19 before returning to control values 2 days after delivery (Figures 2(a) and 2(b)). In addition, the sensitivity of extrasynaptic GABAARs to 3*α*,5*α*-THP (1 *μ*M) resulted more pronounced during the late pregnancy compared to estrus [34] (Figures 3(a) and 3(b)).

## 6. Change in GABAergic System during Pregnancy and Postpartum Period: Implication for Humans

To date, only few studies have focused on understanding the neurochemical mechanisms underlying psychological female status during pregnancy and postpartum period, with particular regard to modifications related to GABAergic inhibition. Hormonal fluctuations may play a key role in controlling mood states in pregnant woman. Several authors suggest that prenatal mood may predict postnatal depression [41, 42] and there is a frequent association between pregnancy-related depressive mood and early gestational age, low birth weight, and premature delivery [43, 44]. During pregnancy, placental tissue synthesizes a large amount of progesterone which exerts both peripheral and central actions. At peripheral level, its action is directed towards reducing maternal immune response [45] and counteracting myometrial contractility [46]. As mentioned previously in this review, the principal effect of progesterone at central level is to interact with GABAARs through its active metabolites that, acting at this level, can regulate a variety of psychological phenomena, including anxiety, sleep, depression, and seizures [47–49]. Although the metabolic pathways of neuroactive steroids and their pronounced modulatory action are well known, only a limited number of investigations have been published that are concerned with the understanding of the effect of their fluctuation during pregnancy and after delivery. For example, while the increase of 3*α*,5*α*-THP levels during pregnancy was observed with no apparent correlation with mood status of pregnant women [21, 22], a direct relationship between intensity of depression and increase of some neuroactive steroid derivatives during pregnancy was suggested [50]. In addition, such modifications were identified in different pathophysiological disorders where the increased seizure susceptibility and anxiety represent a common aspect. The available literature of the last 20 years concerning pregnant women with epilepsy (WWE) reveals an increase in seizure frequency [51] which proves, in part, the complex correlation between pregnancy and epilepsy-related seizures. Conversely, other research groups suggest that pregnancy has variable effects on seizure frequency [52–55].

All these lines of evidence suggest that peripartum period in women could be correlated at least in part to parallel modifications in steroids levels as well as GABAergic system and its components, but some of these alterations need a more deep evaluation in order to find a clear correlation between these aspects and more specific drug treatment needed to counteract some drastic changes in mood during pregnancy and postpartum period.

## 7. Conclusions

The data reported in this review describe the changes in expression and function specific GABAARs in the rat brain during pregnancy and postpartum period. Our data, together with results from other studies [25, 33, 34, 56–58], support the idea that the fluctuations of neuroactive steroid, during such critical periods, are causally related to the observed GABAAR plasticity.

The marked increase of 3*α*,5*α*-THP in the brain that occurs mainly in the late phase of pregnancy (P15–P19) is associated with parallel downregulation of the *γ*2 subunit of the GABAAR in the cerebral cortex and hippocampus [25, 33]. Furthermore, this decrease in *γ*2 subunit does not support the change in synaptic GABAAR function in individual granule cells of the dentate gyrus, evaluated by whole-cell patch-clamp recording [34]. The latest finding may suggest that decrease in pools of receptors containing the *γ*2 subunit does not influence significantly the changes in synaptic inhibition, at least onto dentate gyrus inhibitory synapses. In agreement with this evidence, expression of gephyrin, a scaffold protein involved in the assembly of synaptic GABAAR clusters and in the plasticity of synaptic receptors, resulted unchanged during pregnancy and after delivery [59] even though the expression of the *γ*2 subunit is reduced [25, 33]. In addition, no significant changes in abundance of other GABAAR subunits mRNA, such as *α*1, *α*2, *α*3, *β*1, *β*2, and *β*3, were detected in the same brain region during pregnancy or after delivery [25, 33], suggesting that the modification of the GABAergic system during pregnancy and postpartum period is mainly directed to extrasynaptic receptors in the hippocampus.

Maguire and colleagues also reported downregulation of the *γ*2 subunit during diestrus of the menstrual cycle with no changes in synaptic currents recorded in granule cells of the dentate gyrus [56]. On the other hand, GABAARs involved in the tonic current as well as the subunit directly responsible for such conductance, such as *α*4 and *δ* subunits, undergo pronounced changes during pregnancy and/or after delivery.

We have shown that late pregnancy is associated with upregulation of the *δ* subunit of the GABAAR accompanied with an increase of tonic currents mediated by extrasynaptic GABAARs in granule cells of the dentate gyrus. Such increase in GABAergic tonic inhibition at P19 may be crucial to counteract the increased excitability and anxiety levels peculiar of the final phase of pregnancy immediately before parturition [60–62]. We also found that expression of the *α*4 subunit of the GABAAR in the hippocampus did not change during pregnancy but increased markedly after delivery where levels of *δ* subunits are still high. Interestingly, a similar pattern of changes of the *α*4 subunit is similar during prolonged treatment and subsequent withdrawal of neuroactive steroids in pharmacological studies [63–66], suggesting that modifications in *α*4 subunit expression may reflect a sudden decrease of neuroactive steroids which may exert their anxiolytic effect during late pregnancy. Thus, during the late phase of pregnancy, an increased density of extrasynaptic *α*4*βδ* receptors with a parallel increase of tonic currents may be important as a mechanism for balancing the physiologic increase of excitability.

Conversely, the postpartum period is characterized by a receptor switch with an increased surface expression of *α*4*βγ*2 receptors that may determine reduction in GABAergic inhibition (presumably due to their faster kinetics) and enhanced neuronal excitability with a parallel increase on anxiety levels [64, 65, 67]. Our results differ from those described in the study by Maguire and Mody, where the expression of both *γ*2 and *δ* subunits in the hippocampus was decreased at P18 compared with that in virgin mice in diestrus [68]. This inconsistency of results may depend on the different species studied and diverse experimental conditions.

In addition, a recent study has suggested that pregnancy can be related to perturbations in *γ* oscillations in the hippocampus through a direct effect on GABAergic synapses onto specific parvalbumin interneurons expressing GABAAR containing the *δ* subunit [69].

All these results provide further evidence of the notion that both expression and function of GABAARs in the brain are regulated during pregnancy and immediately after delivery in response to the marked fluctuations in the brain levels of neuroactive steroids. The high sensitivity of receptors containing the *δ* subunits towards the action of endogenous compounds such as 3*α*,5*α*-THP [13, 70, 71] may support the change in expression of this subunit. Moreover, the expression of *δ* subunit is accompanied by similar and parallel changes in *α*4 subunit [56, 63–65, 72–74] that make GABAARs responsible for tonic inhibition.

The *α*4 subunit is able to form receptors with either *δ* or *γ*2 subunits [75] which are characterized by different function, pharmacology, and synaptic location. During pregnancy, there is an increased expression of the *δ* subunit with an enhanced function of extrasynaptic GABAAR apparent in granule cells of the dentate gyrus, while there is a parallel decrease in the expression of the *γ*2 subunit, with no change in that of the *α*4 subunit. These concomitant events suggest that during pregnancy an increase of *α*4*βδ* is accompanied by a parallel decreased assembly of *α*4*βγ*2 receptors, a pattern similar to that occurring during the ovarian cycle [56]. Consistent with the nature of pregnancy and associated mood states, it is conceivable that a possible role for these events could be that an increase in *α*4*βδ* may be related to an increase in inhibition, while an increase in *α*4*βγ*2 could be associated with an increase in anxiety. A fundamental role in the production of neuroactive steroids and their fluctuations during physiological periods such as pregnancy is played by the enzymes involved in their synthesis. Finasteride, which prevents the synthesis of 3*α*,5*α*-THP through the blockade of 5*α*-reductase, one of the enzymes involved in the synthesis of the 3*α*,5*α*-THP, was used to clarify the effect of neuroactive steroids in the plasticity of GABAARs during pregnancy and after delivery [25, 76, 77]. Finasteride, administered to pregnant rats, prevented both the downregulation of *γ*2 subunit and the upregulation of *δ* subunit observed in the dentate gyrus and CA1 region at P19 [25, 33, 34]. Parallel to these observations, the increase of tonic currents at P19 was also significantly inhibited by finasteride treatment [34]. In contrast, finasteride had no effect on the kinetic properties of GABAergic sIPSCs in granule cells of rats at P19.

The lines of evidence summarized in this review suggest that alterations in the GABAergic system, such as modification of specific subunits and the altered function of certain GABAARs, may result in altered synaptic transmission.

All these findings may prompt future studies directed to increase the knowledge about the physiological alteration of inhibitory synaptic transmission observed in animal models of pregnancy and postpartum period that, in turn, may contribute further to the understanding of the neurochemical changes to the peripartum period in women.

## References

[1] Barnard E. A., Skolnick P., Olsen R. W. (1998). International Union of Pharmacology. XV. Subtypes of *γ*-aminobutyric acidA receptors: classification on the basis of subunit structure and receptor function. *Pharmacological Reviews*.

[2] Nusser Z., Roberts J. D. B., Baude A., Richards J. G., Somogyi P. (1995). Relative densities of synaptic and extrasynaptic GABA_A_ receptors on cerebellar granule cells as determined by a quantitative immunogold method. *Journal of Neuroscience*.

[3] Glykys J., Mody I. (2007). Activation of GABA_A_ receptors: views from outside the synaptic cleft. *Neuron*.

[4] Glykys J., Mann E. O., Mody I. (2008). Which GABAA receptor subunits are necessary for tonic inhibition in the hippocampus?. *The Journal of Neuroscience*.

[5] Semyanov A., Walker M. C., Kullmann D. M., Silver R. A. (2004). Tonically active GABAA receptors: modulating gain and maintaining the tone. *Trends in Neurosciences*.

[6] Farrant M., Nusser Z. (2005). Variations on an inhibitory theme: phasic and tonic activation of GABAA receptors. *Nature Reviews Neuroscience*.

[7] Porcello D. M., Huntsman M. M., Mihalek R. M., Homanics G. E., Huguenard J. R. (2003). Intact synaptic GABAergic inhibition and altered neurosteroid modulation of thalamic relay neurons in mice lacking *δ* subunit. *Journal of Neurophysiology*.

[8] Stell B. M., Brickley S. G., Tang C. Y., Farrant M., Mody I. (2003). Neuroactive steroids reduce neuronal excitability by selectively enhancing tonic inhibition mediated by delta subunit-containing GABA_A_ receptors. *Proceedings of the National Academy of Sciences of the United States of America*.

[9] Wei W., Zhang N., Peng Z., Houser C. R., Mody I. (2003). Perisynaptic cocalization of delta subunit-containing GABAA receptors and their activation by GABA spillover in the mouse dentate gyrus. *The Journal of Neuroscience*.

[10] Jia F., Pignataro L., Schofield C. M., Yue M., Harrison N. L., Goldstein P. A. (2005). An extrasynaptic GABA_A_ receptor mediates tonic inhibition in thalamic VB neurons. *Journal of Neurophysiology*.

[11] Drasbek K. R., Jensen K. (2006). THIP, a hypnotic and antinociceptive drug, enhances an extrasynaptic GABAA receptor-mediated conductance in mouse neocortex. *Cerebral Cortex*.

[12] Glykys J., Mody I. (2006). Hippocampal network hyperactivity after selective reduction of tonic inhibition in GABAA receptor *α*5 subunit-deficient mice. *Journal of Neurophysiology*.

[13] Wohlfarth K. M., Bianchi M. T., Macdonald R. L. (2002). Enhanced neurosteroid potentiation of ternary GABAA receptors containing the delta subunit. *The Journal of Neuroscience*.

[14] Bianchi M. T., Macdonald R. L. (2003). Neurosteroids shift partial agonist activation of GABA_A_ receptor channels from low- to high-efficacy gating patterns. *Journal of Neuroscience*.

[15] Penning T. M., Sharp R. B., Krieger N. R. (1985). Purification and properties of 3*α*-hydroxysteroid dehydrogenase from rat brain cytosol: inhibition by nonsteroidal anti-inflammatory drugs and progestins. *The Journal of Biological Chemistry*.

[16] Russell D. W., Wilson J. D. (1994). Steroid 5alpha-reductase: two genes/two enzymes. *Annual Review of Biochemistry*.

[17] Follesa P., Concas A., Porcu P. (2001). Role of allopregnanolone in regulation of GABA_A_ receptor plasticity during long-term exposure to and withdrawal from progesterone. *Brain Research Reviews*.

[18] Brussaard A. B., Kits K. S., Baker R. E. (1997). Plasticity in fast synaptic inhibition of adult oxytocin neurons caused by switch in GABA_A_ receptor subunit expression. *Neuron*.

[19] Lambert J. J., Cooper M. A., Simmons R. D. J., Weir C. J., Belelli D. (2009). Neurosteroids: endogenous allosteric modulators of GABA_A_ receptors. *Psychoneuroendocrinology*.

[20] Mostallino M. C., Sanna E., Concas A., Biggio G., Follesa P. (2009). Plasticity and function of extrasynaptic GABAA receptors during pregnancy and after delivery. *Psychoneuroendrocrinology*.

[21] Luisi S., Petraglia F., Benedetto C. (2000). Serum allopregnanolone levels in pregnant women: changes during pregnancy, at delivery, and in hypertensive patients. *Journal of Clinical Endocrinology and Metabolism*.

[22] Paoletti A. M., Romagnino S., Contu R. (2006). Observational study on the stability of the psychological status during normal pregnancy and increased blood levels of neuroactive steroids with GABA-A receptor agonist activity. *Psychoneuroendocrinology*.

[23] Milewich L., Gant N. F., Schwarz B. E., Chen G. T., MacDonald P. C. (1979). 5*α*-reductase activity in human placenta. *American Journal of Obstetrics and Gynecology*.

[24] Buster J. E. (1983). Gestational changes in steroid hormone biosynthesis, secretion, metabolism, and action. *Clinics in Perinatology*.

[25] Concas A., Mostallino M. C., Porcu P. (1998). Role of brain allopregnanolone in the plasticity of *γ*-aminobutyric acid type A receptor in rat brain during pregnancy and after delivery. *Proceedings of the National Academy of Sciences of the United States of America*.

[26] Baulieu É.-É. (1991). Neurosteroids: a new function in the brain. *Biology of the Cell*.

[27] Sanna E., Talani G., Busonero F. (2004). Brain steroidogenesis mediates ethanol modulation of GABA_A_ receptor activity in rat hippocampus. *Journal of Neuroscience*.

[28] Izumi Y., Murayama K., Tokuda K., Krishnan K., Covey D. F., Zorumski C. F. (2007). GABAergic neurosteroids mediate the effects of ethanol on long-term potentiation in rat hippocampal slices. *European Journal of Neuroscience*.

[29] Maayan R., Fisch B., Galdor M. (2004). Influence of 17*β*-estradiol on the synthesis of reduced neurosteroids in the brain (in vivo) and in glioma cells (in vitro): possible relevance to mental disorders in women. *Brain Research*.

[30] Belelli D., Lambert J. J. (2005). Neurosteroids: Endogenous regulators of the GABA_A_ receptor. *Nature Reviews Neuroscience*.

[31] Majewska M. D. (1992). Neurosteroids: Endogenous bimodal modulators of the GABAA receptor. Mechanism of action and physiological significance. *Progress in Neurobiology*.

[32] Staley K., Scharfman H. (2005). A woman's prerogative. *Nature Neuroscience*.

[33] Follesa P., Floris S., Tuligi G., Mostallino M. C., Concas A., Biggio G. (1998). Molecular and functional adaptation of the GABA_A_ receptor complex during pregnancy and after delivery in the rat brain. *European Journal of Neuroscience*.

[34] Sanna E., Mostallino M. C., Murru L. (2009). Changes in expression and function of extrasynaptic GABA_A_ receptors in the rat hippocampus during pregnancy and after delivery. *Journal of Neuroscience*.

[35] Concas A., Follesa P., Barbaccia M. L., Purdy R. H., Biggio G. (1999). Physiological modulation of GABA_A_ receptor plasticity by progesterone metabolites. *European Journal of Pharmacology*.

[36] Pirker S., Schwarzer C., Wieselthaler A., Sieghart W., Sperk G. (2000). GABA_A_ receptors: immunocytochemical distribution of 13 subunits in the adult rat brain. *Neuroscience*.

[37] Knoflach F., Benke D., Wang Y. (1996). Pharmacological modulation of the diazepam-insensitive recombinant gamma-aminobutyric acidA receptors alpha 4 beta 2 gamma 2 and alpha 6 beta 2 gamma 2. *Molecular Pharmacology*.

[38] Wafford K. A., Thompson S. A., Thomas D., Sikela J., Wilcox A. S., Whiting P. J. (1996). Functional characterization of human gamma-aminobutyric acidA receptors containing the alpha 4 subunit. *Molecular Pharmacology*.

[39] Nusser Z., Mody I. (2002). Selective modulation of tonic and phasic inhibitions in dentate gyrus granule cells. *Journal of Neurophysiology*.

[40] Caraiscos V. B., Elliott E. M., You-Ten K. E. (2004). Tonic inhibition in mouse hippocampal CA1 pyramidal neurons is mediated by *α*5 subunit-containing *γ*-aminobutyric acid type A receptors. *Proceedings of the National Academy of Sciences of the United States of America*.

[41] Green J. M. (1990). ‘Who is unhappy after childbirth?’: antenatal and intrapartum correlates from a prospective study. *Journal of Reproductive and Infant Psychology*.

[42] Hobfoll S. E., Ritter C., Lavin J., Hulsizer M. R., Cameron R. P. (1995). Depression prevalence and incidence among inner-city pregnant and postpartum women. *Journal of Consulting and Clinical Psychology*.

[43] Hedegaard M., Brink Henriksen T., Sabroe S., Secher N. J. (1993). Psychological distress in pregnancy and preterm delivery. *British Medical Journal*.

[44] Teixeira J. M. A., Glover V., Fisk N. M. (1999). Acute cerebral redistribution in response to invasive procedures in the human fetus. *American Journal of Obstetrics and Gynecology*.

[45] Ragusa A., de Carolis C., dal Lago A. (2004). Progesterone supplement in pregnancy: an immunologic therapy?. *Lupus*.

[46] Yallampalli C., Gangula P. R. R., Kondapaka S., Fang L., Wimalawansa S. (1999). Regulation of calcitonin gene-related peptide receptors in the rat uterus during pregnancy and labor and by progesterone. *Biology of Reproduction*.

[47] Wang M., Seippel L., Purdy R. H., Bäckström T. (1996). Relationship between symptom severity and steroid variation in women with premenstrual syndrome: study on serum pregnenolone, pregnenolone sulfate, 5alpha-pregnane-3,20- dione and 3alpha-hydroxy-5alpha-pregnan-20-one. *Journal of Clinical Endocrinology and Metabolism*.

[48] Uzunova V., Sheline Y., Davis J. M. (1998). Increase in the cerebrospinal fluid content of neurosteroids in patients with unipolar major depression who are receiving fluoxetine or fluvoxamine. *Proceedings of the National Academy of Sciences of the United States of America*.

[49] Ströhle A., Romeo E., Hermann B. (1999). Concentrations of 3*α*-reduced neuroactive steroids and their precursors in plasma of patients with major depression and after clinical recovery. *Biological Psychiatry*.

[50] Murphy B. E. P., Steinberg S. I., Hu F.-Y., Allison C. M. (2001). Neuroactive ring A-reduced metabolites of progesterone in human plasma during pregnancy: elevated levels of 5*α*-dihydroprogesterone in depressed patients during the latter half of pregnancy. *Journal of Clinical Endocrinology and Metabolism*.

[51] Schmidt A. (1982). The effect of pregnancy on the natural history of epilepsy: review of the literature. *Epilepsy, Pregnancy, and the Child*.

[52] Sabers A., A'Rogvi-Hansen B., Dam M. (1998). Pregnancy and epilepsy: a retrospective study of 151 pregnancies. *Acta Neurologica Scandinavica*.

[53] Rück J., Bauer J. (2008). Pregnancy and epilepsy. Retrospective analysis of 118 patients. *Der Nervenarzt*.

[54] Sveberg L., Svalheim S., Taubøll E. (2015). The impact of seizures on pregnancy and delivery. *Seizure*.

[55] Taubøll A., Sveberg L., Svalheim S. (2015). Interactions between hormones and epilepsy. *Seizure*.

[56] Maguire J. L., Stell B. M., Rafizadeh M., Mody I. (2005). Ovarian cycle-linked changes in GABA_A_ receptors mediating tonic inhibition alter seizure susceptibility and anxiety. *Nature Neuroscience*.

[57] Fénelon V. S., Herbison A. E. (1996). Plasticity in GABA_A_ receptor subunit mRNA expression by hypothalamic magnocellular neurons in the adult rat. *Journal of Neuroscience*.

[58] Brussaard A. B., Koksma J.-J. (2003). Conditional regulation of neurosteroid sensitivity of GABA_A_ receptors. *Annals of the New York Academy of Sciences*.

[59] Sassoè-Pognetto M., Follesa P., Panzanelli P. (2007). Fluctuations in brain concentrations of neurosteroids are not associated to changes in gephyrin levels. *Brain Research*.

[60] Zuluaga M. J., Agrati D., Pereira M., Uriarte N., Fernández-Guasti A., Ferreira A. (2005). Experimental anxiety in the black and white model in cycling, pregnant and lactating rats. *Physiology and Behavior*.

[61] de Brito Faturi A., Teixeira-Silva F., Leite J. R. (2006). The anxiolytic effect of pregnancy in rats is reversed by finasteride. *Pharmacology Biochemistry and Behavior*.

[62] Skouteris H., Wertheim E. H., Rallis S., Milgrom J., Paxton S. J. (2009). Depression and anxiety through pregnancy and the early postpartum: an examination of prospective relationships. *Journal of Affective Disorders*.

[63] Sundstrom-Poromaa I., Smith D. H., Gong Q. H. (2002). Hormonally regulated *α*
_4_
*β*
_2_
*δ*GABA_A_ receptors are a target for alcohol. *Nature Neuroscience*.

[64] Smith S. S., Gong Q. H., Hsu F.-C., Markowitz R. S., Ffrench-Mullen J. M. H., Li X. (1998). GABA_A_ receptor *α*4 subunit suppression prevents withdrawal properties of an endogenous steroid. *Nature*.

[65] Smith S. S., Gong Q. H., Li X. (1998). Withdrawal from 3a-OH-5a-pregnan-20-one using a pseudopregnancy model alters the kinetics of hippocampal GABAA-gated current and increases the GABAA receptor a4 subunit in association with increased anxiety. *The Journal of Neuroscience*.

[66] Biggio F., Gorini G., Caria S. (2006). Plastic neuronal changes in GABA_A_ receptor gene expression induced by progesterone metabolites: in vitro molecular and functional studies. *Pharmacology Biochemistry and Behavior*.

[67] Smith S. S., Ruderman Y., Frye C., Homanics G., Yuan M. (2006). Steroid withdrawal in the mouse results in anxiogenic effects of 3*α*,5*β*-THP: a possible model of premenstrual dysphoric disorder. *Psychopharmacology*.

[68] Maguire J., Mody I. (2008). GABA_A_R plasticity during pregnancy: relevance to postpartum depression. *Neuron*.

[69] Ferando I., Mody I. (2013). Altered gamma oscillations during pregnancy through loss of *δ* subunit-containing GABA_A_ receptors on parvalbumin interneurons. *Frontiers in Neural Circuits*.

[70] Adkins C. E., Pillai G. V., Kerby J. (2001). Alpha4beta3delta GABA_A_ receptors characterized by fluorescence resonance energy transfer-derived measurements of membrane potential. *The Journal of Biological Chemistry*.

[71] Brown N., Kerby J., Bonnert T. P., Whiting P. J., Wafford K. A. (2002). Pharmacological characterization of a novel cell line expressing human **α**
_4_
**β**
_3_
**δ**GABA_A_ receptors. *British Journal of Pharmacology*.

[72] Griffiths J. L., Lovick T. A. (2005). GABAergic neurones in the rat periaqueductal grey matter express *α*4, *β*1 and *δ*GABA_A_ receptor subunits: plasticity of expression during the estrous cycle. *Neuroscience*.

[73] Lovick T. A., Griffiths J. L., Dunn S. M. J., Martin I. L. (2005). Changes in GABA_A_ receptor subunit expression in the midbrain during the oestrous cycle in Wistar rats. *Neuroscience*.

[74] Shen H., Gong Q. H., Yuan M., Smith S. S. (2005). Short-term steroid treatment increases *δ*GABA_A_ receptor subunit expression in rat CA1 hippocampus: pharmacological and behavioral effects. *Neuropharmacology*.

[75] Whiting P. J., Bonnert T. P., McKernan R. M. (1999). Molecular and functional diversity of the expanding GABA-A receptor gene family. *Annals of the New York Academy of Sciences*.

[76] Azzolina B., Ellsworth K., Andersson S., Geissler W., Bull H. G., Harris G. S. (1997). Inhibition of rat *α*-reductases by finasteride: evidence for isozyme differences in the mechanism ofinhibition. *Journal of Steroid Biochemistry and Molecular Biology*.

[77] Finn D. A., Beadles-Bohling A. S., Beckley E. H. (2006). A new look at the 5*α*-reductase inhibitor finasteride. *CNS Drug Reviews*.

